# Potential quality pitfalls of digitalized whole slide image of breast pathology in routine practice

**DOI:** 10.1038/s41379-021-01000-8

**Published:** 2021-12-27

**Authors:** Nehal M. Atallah, Michael S. Toss, Clare Verrill, Manuel Salto-Tellez, David Snead, Emad A. Rakha

**Affiliations:** 1 Department of Histopathology, School of Medicine, the University of Nottingham and Nottingham University Hospitals NHS Trust, Nottingham, UK; 2 Department of Pathology, Faculty of Medicine, Menoufia University, Shebin Elkom, Al-Menoufia, Egypt; 3 Nuffield Department of Surgical Sciences, University of Oxford, Oxford, UK; 4 NIHR Oxford Biomedical Research Centre, University of Oxford, John Radcliffe Hospital, Oxford, UK; 5 Precision Medicine Centre of Excellence, The Patrick G Johnston Centre for Cancer Research, Queen’s University, Belfast, UK; 6 Cellular Pathology, University Hospitals Coventry and Warwickshire NHS Trust, Coventry, Coventry, UK

**Keywords:** Cancer, Breast cancer

## Abstract

Using digitalized whole slide images (WSI) in routine histopathology practice is a revolutionary technology. This study aims to assess the clinical impacts of WSI quality and representation of the corresponding glass slides. 40,160 breast WSIs were examined and compared with their corresponding glass slides. The presence, frequency, location, tissue type, and the clinical impacts of missing tissue were assessed. Scanning time, type of the specimens, time to WSIs implementation, and quality control (QC) measures were also considered. The frequency of missing tissue ranged from 2% to 19%. The area size of the missed tissue ranged from 1–70%. In most cases (>75%), the missing tissue area size was <10% and peripherally located. In all cases the missed tissue was fat with or without small entrapped normal breast parenchyma. No missing tissue was identified in WSIs of the core biopsy specimens. QC measures improved images quality and reduced WSI failure rates by seven-fold. A negative linear correlation between the frequency of missing tissue and both the scanning time and the image file size was observed (*p* < 0.05). None of the WSI with missing tissues resulted in a change in the final diagnosis. Missing tissue on breast WSI is observed but with variable frequency and little diagnostic consequence. Balancing between WSI quality and scanning time/image file size should be considered and pathology laboratories should undertake their own assessments of risk and provide the relevant mitigations with the appropriate level of caution.

## Introduction

The introduction of digitalized whole slide image (WSI) technology has attracted a great deal of attention in the pathologists’ community where the use of digital pathology (DP) for primary diagnosis is now becoming commonplace^1–4^. Currently, several pathology laboratories are either fully or partially digitalized, and various governmental funds are provided to increase the adoption of this technology accelerated by the recent Food and Drug Administration (FDA) approval of WSI devices for primary diagnosis^5^. The introduction of this paradigm shift in technology allows not only remote reporting with flexible working hours, persistent service delivery even in challenging situations such as the COVID-19 pandemic, second and expert opinion reporting^6^ but also provide a platform in which artificial intelligence (AI)-based algorithms to be implemented.

Digitizing an entire glass slide is a complex multistep process that depends on the integration of state‑of‑the‑art scanner hardware, robotics, and software. Producing optimal quality WSIs also depends on the skills of a well‑trained operator who guides the scanning procedure in addition to robust quality control (QC) measures starting from receiving the specimen through fixation, processing, and slide preparation prior to the actual scanning process and acquisition of the WSIs^7^. The representation of the tissue within the WSI compared to the corresponding glass slides is of crucial importance for the adoption of DP in routine practice for making an accurate diagnosis and sharing challenging cases for a second opinion. Archiving both glass slides and WSIs in routine practice is not cost effective and will require extra resources, however, it is unlikely that pathology laboratories will abandon storing glass slides and rely on the archived the digitized WSI unless there is evidence-based reassurance that WSI is fully representative of the corresponding glass slides. Such evidence(s) should be strong enough to change the national guidelines. Furthermore, to facilitate slide scanning at a large scale in routine histopathology reporting, it is desirable for scanner manufacturers to provide fast high throughput scanners able to deal with the volume of slides produced by modern laboratories, whilst maintaining the image quality. A largely automated scanning process is required to deliver this, and it important such automation avoids scanning errors from missing low-density tissue such as adipose tissue.

In this study, we aimed to assess the representation of digitized WSIs when compared to the corresponding glass slides and the associated clinical implication. We have used histopathology WSIs acquired from various breast lesions, as a model of fatty tissue rich organ, which is the most problematic tissue to be scanned due to its pale nature with reduced contrast and features a spectrum of pathological lesions and tissue densities. This was evaluated across multiple breast specimen types and scanning protocols with emphasize on the role of QC measures in minimizing these pitfalls.

## Material and methods

This study included a total of 40,160 WSIs including breast resections and biopsies. The study cohort included 2 groups as follows:

### The main study cohort (Group 1)

This cohort comprised 22,160 WSIs (22,000 Hematoxylin and Eosin (H&E) images and 160 images from immunohistochemistry (IHC) stained slides) scanned by the Philips Intellisite UltraFast Scanner, (Amsterdam, Netherland) (Scanner A) from breast patients presented to Nottingham University Hospitals, Nottingham, United Kingdom (UK) in the period between March 2020 to January 2021. This cohort included a wide range of breast excisions including normal tissue from reduction and re-excision specimen, benign lesions, atypical proliferation, and malignant lesions either in situ or invasive tumors.

To assess the frequency of missing tissue in WSIs scanned for research purposes and to assess the impact of using different scanning approaches (with or without human input during scanning) on the frequency of missing tissue in the digitalized slides, a different cohort (Group 2) was used. This cohort included 18,000 historical WSIs of invasive breast carcinoma (BC) that were scanned for various research purposes as part of the Nottingham Breast Pathology Research Group activity. The WSIs in this cohort comprised 10,000 images acquired from Aperio AT2—High Volume Scanner (Leica Microsystems (UK) Ltd) (Scanner B) while the remaining 8,000 WSIs were scanned on Pannoramic 250 FLASH III 2.0 (3DHistech, Budapest, Hungary) (Scanner C). This cohort was used to study the frequency of missing tissue in WSIs produced by different scanning approaches (with or without human input during scanning) and to assess the clinical impacts of missing tissue on tumor and resection margin assessments. The human input in this cohort is represented in manual orientation and outline annotation of the focus points in scanner B and changing specimen threshold settings in scanner C. The setting in the specimen threshold level ranges between 0 and 255. The default value is 220. Smaller values (low threshold) result in more sensitive scanning, with more areas to be captured but also more time and larger file size. High threshold values result in a less sensitive faster scanning.

All scanning was carried out at ×40 magnification equivalent to 0.25 μm/pixel resolution. WSIs were visualized by specific viewer software (Philips Image Management System IMS, Aperio image scope version 12.4.3 and 3DHistech case viewer Version 2.4), respectively. In each image we have assessed missing tissue frequency, the image quality and resolution and the role of applying various QC measures in lowering the WSI rejection rate.

### Comparison of the missing tissue between glass slide and WSIs

We compared each WSI with its corresponding glass slides. The image thumbnail (the low-resolution whole section view) was used to evaluate whether the missed tissue was obvious and easily detected by the pathologist or not. In each image the missing tissue was assessed regarding; (1) The percentage of the missed tissue in relation to the whole tissue (percentage of missing tissue = $\frac{{{{{{{\rm{area}}}}}}\,{{{{{\rm{of}}}}}}\,{{{{{\rm{missed}}}}}}\,{{{{{\rm{tissue}}}}}}\,{{{{{\rm{in}}}}}}\,{{{{{\rm{mm}}}}}}}}{{{{{{{\rm{total}}}}}}\,{{{{{\rm{tissue}}}}}}\,{{{{{\rm{aera}}}}}}\,{{{{{\rm{in}}}}}}\,{{{{{\rm{mm}}}}}}}}$) Multifocal missed tissue areas were summed up and the final total percentage was estimated. (2) Missing tissue type i.e., fatty tissue, normal breast lobules and ducts or any pathological lesions. The latter was classified into invasive tumor and its type, in situ components and atypical proliferative lesions including ductal and lobular, and benign lesions such as intraductal papilloma, fibroadenoma and radial scar. (3) The site or location of the missed tissue in relation to the whole tissue section (peripheral, central, or multifocal). (4) Confounders including specimen types (e.g., excision specimens, or core biopsy), the relation between time of the implementation of the digitalization service and missing tissue frequency and the type of stain. in addition, the relation between the frequency of missing tissue and scanning time and image file size was evaluated using the three scanners.

### WSI quality control (QC)

Data on the scanned digital image failure rates before and after the application of QC measures performed during routine scanning were collected. WSI QC included macro-evaluation of pre-analytic slide artefacts that should have been resolved before scanning. Protocols were applied for glass slide QC including glass slides should be intact (not broken or cracked), appropriately stained and dried, clean without any ink markings, properly placed coverslips without hanging over the edge of the glass slide, and air bubbles were to be absent; the glass slide label should be flat and not extending past the slide edge or covering tissue on the slides. Small air bubbles over the tissue section or varied size air bubbles away from the tissue section are usually acceptable in examining slides using the conventional microscope as they do not affect the morphology. However, during WSIs acquisition using digital scanners, these air bubbles produce uneven slide surface and hence the produced image usually shows areas of tissue out of focus which will affect the overall WSI quality. Additional real-time QC was performed by the scanner that provided user interface messages including barcode detection failures, no tissue detection, macro focus image failure, or image quality errors. Post-scan QC measure included a review of the WSI to visualize utilizing the image thumbnails to ensure all tissue present on the glass slide was scanned. Poor WSI quality resulting from a glass slide quality was an indication for reprocessing of the glass slides (e.g., recutting, remounting, re-staining, or re-placing the coverslip properly) whereas failed scanning of an adequate glass slide during scanning errors such as out of focus or obvious missed tissue was an indication of rescanning.

The clinical significance of the missing tissue and WSI quality was evaluated. The impacts assessed included any change in the recognition of tissue type examined, identification of the lesion, classification of the lesion, tumor type, grade, tumor stage (lymph node metastasis, tumor size), and surgical resection margin distance and completeness of excision. For the clinical significance of the missed tissue on the IHC stained slides, we evaluated the change of Ki67 and PR final score between glass slides and WSI.

### Statistical analysis

Data were collected, tabulated, and statistically analyzed with “statistical package for the social sciences (SPSS) version 24 program. IBM SPSS statistics for windows, version 24.0, NY: IBM Corp.). Descriptive Statistics i.e., Arithmetic mean (x) was used as a measure of central tendency, standard deviation was used as a measure of dispersion, Percentage (%), Range and Median. Fisher’s exact test (FE) and Chi-square (*χ*^2^) were used to compare between qualitative data. Differences were considered statistically significant when (*P* < 0.05).

## Results

### Missing tissue characteristics

**Fig. 1:**
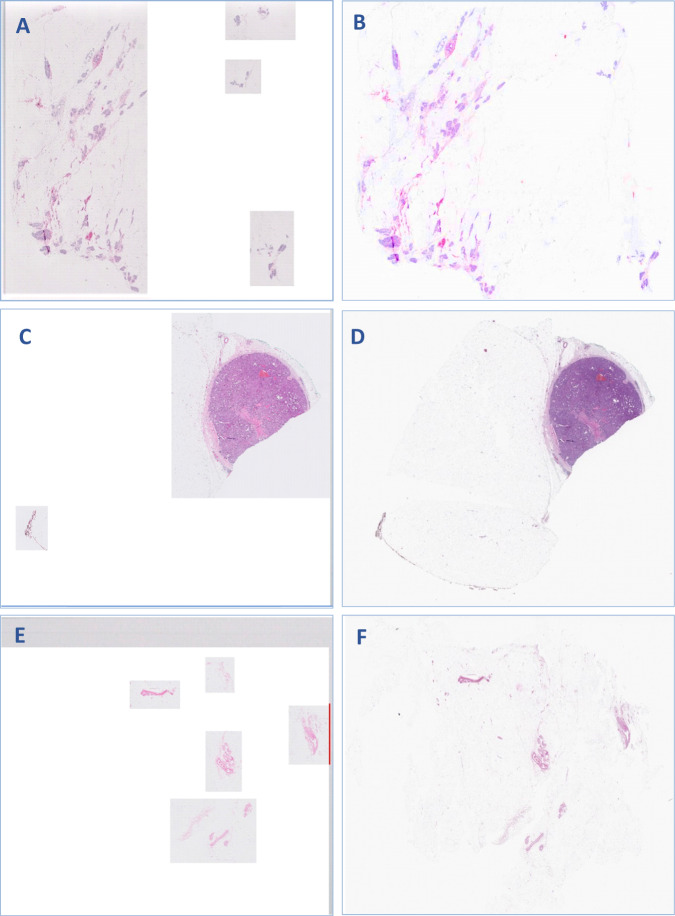
Examples of missed tissue. Examples of missed tissue in the whole slide images (WSI) as shown in (**A**, **C**, **E**) compared with the corresponding glass slides (**B**, **D**, **F**).

**Fig. 2:**
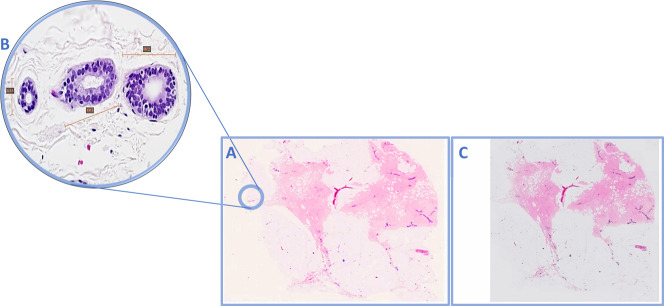
Example of WSI missing normal breast duct at the periphery of the slides. **A** Overview image of a slide that was fully scanned, **B** high power of the normal breast terminal lobular duct unit on glass slide (×40 light microscope) present at periphery of slide and was missed upon scanning (**C**). The terminal lobular duct measured 1.5 mm at largest diameter.

In this study, 19% of all WSIs showed a variable degree of missing tissue and the mean proportion of the missed tissue was 13%, range 1–70% (Table 1). In 49% of cases, the missing tissue was <5% while in the majority of cases (82%), the missing tissue was < 20%. Only 1% showed >50% of tissue loss (Supplementary Table). In all cases, the missed tissue was fibrofatty tissue with or without minute vasculatures (100%). Only 8% of cases showed missed small normal breast ducts and/or lobules, in addition to the missing fatty tissue (Figs. 1 and 2). All the missed ducts were peripherally located and surrounded by fat. The largest missed terminal lobular duct unit (TLDU) was 1.5 mm in maximum diameter and the largest duct that was missed on scanning was measured 830 µm in maximum diameter. The missed tissue was allocated peripherally in 78% of the cases, while 22% of cases showed centrally and peripherally located missed tissue. None of the examined core needle biopsy specimens (*n* = 185) revealed missed tissue when compared to the corresponding glass slides.

**Table 1:** Characteristics of the whole slide images (WSI) scanned in the routine diagnostic practice and showed missing tissues (number = 4268).

Parameters	Cases with missing tissue	
	*n*	%
Type of missed tissue		
Fat and blood vessels	4268	100
Normal Ducts/TDLUs	1760	7.9
Benign lesions	0	0
In situ carcinoma	0	0
Invasive carcinoma	0	0
Site of missed tissue		
Peripheral only	3337	78.2
Central and peripheral	931	21.8
Type of specimens		
Resection specimens	4268	100
Core needle biopsy	185	0
Date of scanning		
Early^a^	2068	48.5
Late	2200	51.5
Change in final diagnosis		
Yes	0	0
No	4268	100

*TDLU* terminal duct lobular unit.

^a^First 3 months after installation and the use of the scanner.

Focusing on WSIs of invasive BC tissue sections only, the mean proportion of missing tissue for the 3 scanners collectively was 9% (range 3–19%). Within group 2 (research setting), the missing tissue frequency was 8% and 3% for scanners C and B, respectively.

None of the WSIs with missing tissues resulted in a change in the final diagnosis i.e., differentiating benign from malignant lesions, change tumor grade or stage, however there was an underestimation of the distance to margins when the missed tissue was peripherally allocated. 12% of cases revealed a reduction of distance to actual margin, and the reduction ranged from 1 to 8 mm. In all cases the margins were inked facilitating the identification of the defects and that a missing part of the tissue present opposite to the tumor, particularly when it was close (1 mm). Importantly, none of the cases was reported as tumor on ink (positive margin) because of missing tissue on WSI thus no subsequent re-excision was performed.

Regarding WSIs of the IHC stained tissues, 16% of the case showed degree of missing tissue. The mean proportion of missed tissue was 6% and ranged between (2–10%). The missed tissue was fat in all cases with occasional normal breast TLDUs. There was no change in the final scoring categories between the glass slide and WSI in PR stained slides. In ki67 stained WSIs, one case (1.25%) showed missing few invasive tumor cells infiltrating fat and resulted in a reduction of the Ki67 score from 15% on a glass slide to 12% on WSI when we considered average ki67 expression in whole slide. However, if the expression is considered on the hotspots, there was no difference on the final scoring category. In either scoring methods, this did not affect the final scoring category of the cases as the cut-off of positivity used is 10%.

### Quality control data

**Fig. 3:**
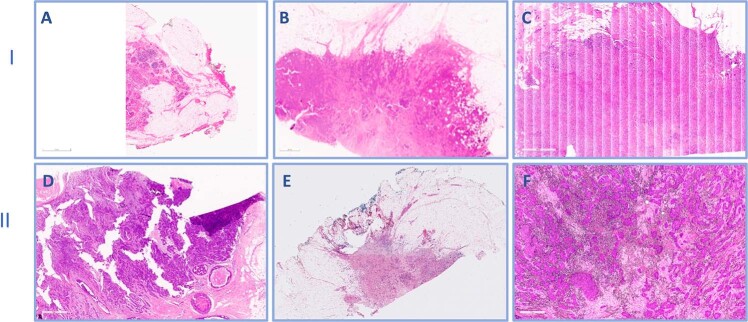
Examples of different quality control (QC) categories on failed whole slide images (WSIs). I: Failed WSI due to scanning errors:**A** Incomplete slide scanning, **B** Out of Focus image, **C** Improper line stitching led to longitudinal scores. II: Failed WSI due to improper slide preparations. **D** Thick sections with tissue cracking and folding, **E** Uneven H&E Stain distribution, **F** Air bubbles on slide.

**Fig. 4:**
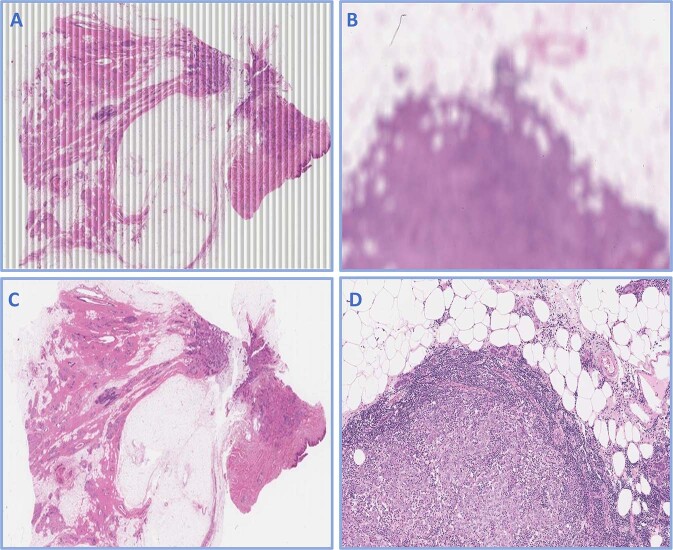
Role of quality control in improving digitalized image quality. Failed images before (**A**, **B**) and after quality control (**C**, **D**). **A** shows longitudinal scores, **B** is out of focus.

**Fig. 5:**
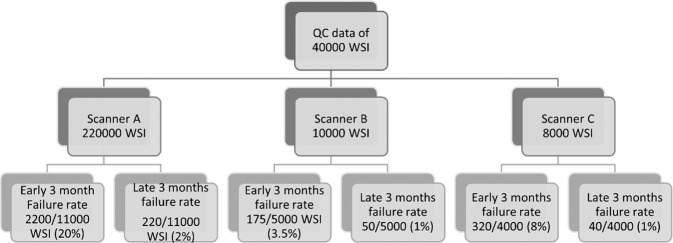
A flowchart demonstrates WSI failure rate before and after application of quality control measures for the three studied scanners.

During the early implementation of the scanning service, the WSI scanning failure rate was 20%. Failed image quality was related to poor quality issues including pixelated or out of focus image or failed scanning (Fig. 3l). Poor quality images that necessitate rescanning partial related to suboptimal glass slide qualities including tissue folding, cracking, uneven stain distribution and/or air bubbles (Fig. 3II). Following 3 months after applying strict QC measures of both glass slides and WSI, the scanned images failure rates decreased to 2% using scanner A (Figs. 4 and 5). Although there was a significant reduction in WSIs failure rate (*p* < 0.05) after applying the QC measures, there was no significant difference in the frequency of missing tissue and the time to implementation of the digitalization service (*p* > 0.05).

### Missing tissue and confounders

**Fig. 6:**
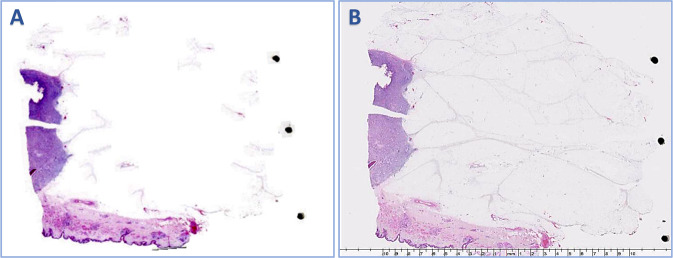
Example of image scanned by the same scanner but at two different settings. **A** High specimen threshold scanning with missing most of the fatty tissue on WSI, while in (**B**), the image was scanned at low specimen threshold scanning and no missing tissue was detected.

The average time consumed to scan each slide by scanner (A) was 60 s (range 30–90 s) and the average image file size was 1.2 GB (range 1.1–1.4 GB) (Table 2). Scanning time for one slide in scanner B was ranging between 7–9 min (average 8 min) including time required for manual orientation and outline annotation of the focus points and the average image size was about 1.5 GB (range 1.4–1.7 GB). For scanner C, when using a low specimen threshold setting, the average scanning time was 2.5 min (range 3–4 min), and the average image file size was 1.7 GB (range 1.5–1.8 GB). For the high specimen threshold setting (time for each slide scanning ranged between 1–2 min and the average image file size was 800 MBs (Fig. 6).

**Table 2:** Relation between missing tissue and both, the scanning time and average image file size with their impact on the clinical final diagnosis.

Studied scanners	Scanning time	Average image file size	Missing tissue/type	Out of focus images	Impact on the final clinical diagnosis
Scanner A	0.5–1.5 min	1.2 GB	Yes/Fat	No	No effect
Scanner B	7-9 min	1.5 GB	No	No	No effect
Scanner C					
• High Specimen threshold	1–2 min	800 MB	Yes/Fat	No	No effect
• Low Specimen threshold	3-4 min	1.7 GB	No	No	No Effect

## Discussion

Validation studies reported that WSI is noninferior to glass slides for routine diagnostic work^8–10^. As the adoption of DP and WSI technology is expanding, standardization and best practice guidelines are inevitable to guarantee the optimal implementation and application of these systems without affection of the patient healthcare^11^.

Rendering an accurate diagnosis using WSI rests on the premise that the image represents an accurate digital reproduction of the scanned glass slide. If diagnostic material is missing in the digital image, compared to its glass slide counterpart, this could result in a misdiagnosis and hence improper patient management. The College of American Pathologists recommendations for validating WSI for diagnostic use is to confirm that all the material present on a glass slide is scanned and could be viewed on the digital image. However, this approach seems impractical as the percentage of missing tissue in WSIs is variable between tissue types, scanners and pre scanning processing. Also, pathologists accept glass slides that show a proportion of missing tissue if compared with the actual tissue that was embedded originally in the paraffin block, and they can report the cases based on such slides without ordering recuts as long as the slides are interpreted together and in context of the individual case circumstances. Therefore, seeking strict criteria to accept WSIs usage in routine practice will affect the implementation of such promising technology and prevent the privilege that it can provide to the healthcare service. Thus, the best approach is to provide evidence-based studies showing the differences between glass slides and WSIs and how these differences (if any) can affect the clinical decisions.

Although many studies have focused on validating the diagnostic concordance of WSI and light microscope^12^, few have discussed the representation of the glass slide into digital image, and its impact on the clinical decision making.

In this study, we aimed to assess the quality of WSI performance in routine practice, through assessment of different variables including the frequency, nature, and location of missing tissue on WSI compared to glass slides and its clinical implications. Image quality and the role of QC measures in minimizing these pitfalls was also addressed.

The rationale behind using the breast as a model is that it is formed normally of various tissue types and has a wide spectrum of heterogeneous lesions and pathological entities that covers a considerable sector in pathology i.e. Well differentiated breast cancer that is mostly glandular could represent gastric tissue, liposarcoma as an example of soft tissue tumors, metaplastic carcinoma as a model of squamous cell carcinoma. It also contains a considerable amount of fatty tissue which is well known to be difficulty captured by the digital scanners due to its pale nature.

The frequency of missing tissue was variable among the various cohorts and scanners used in the study. Although direct comparison between the cohorts and the scanner has limitations due to lack of randomization, this variation can be related to difference in the composition of the scanned cohort whether it is mostly faint loose fibrofatty tissue or dense tissue. The first cohort was routine clinical practice cases where each case contains sections from tumor tissue, normal breast tissue, margins, and various types of lesions while second cohort included sections from invasive tumor only which were selected based on the tumor burden and thus less fatty tissue. The latter cohort was scanned in research setting with different scanning protocols based on the research activities. So, cohort 1 was expected to have more fatty tissue component than cohort 2. Moreover, the in-built functionalities and mechanisms that the scanner uses during capturing the WSIs are different. Automatic tissue finder algorithms scanner differs from others with human inputs. Fatty tissue was the most frequent tissue to be missed. Fatty tissue is less dense and fainter than the surrounding tissues, so it is easily to be missed by the scanner detection system especially in thinner tissue sections and paler staining density^9,13^. Also, lipocytes are vacuolated empty spaces which are separated by thin cell membranes/septae so they could be considered as the empty slide white background and hence the scanner camera skips these areas. This could explain why the scanner can capture other faintly stained tissues/structures like mucin pool in mucinous carcinoma for instance as they usually have a bit of color contrast compared to the background slide white spaces. None of mucinous carcinomas included in this study showed missed tissue. The small proportion of WSIs with missed TDLU were at the periphery of the tissue section and were surrounded by fatty tissue and these ducts were minute and widely spaced which could explain the reason of being missed.

The incidence of missing a tissue was higher when it was peripherally allocated than being centrally present. This could be explained by both the mechanism at which the scanner works and the specimen itself. Scanners with automated algorithms to detect tissues tend to neglect peripherally faint and loose tissue to speed up the scanning process. Also, operators may ignore peripheral fatty tissue during setting the scanning area (cropping) and the focus points to reduce the scanning time and image file size. Peripherally situated tissues in larger tissue sections that extend beyond the coverslip are prone to incomplete scanning than fatty tissue present centrally. Furthermore, fatty stroma among invasive BC is usually infiltrated by the malignant cells and consequently easily detected and less likely to be missed.

Importantly, no pathological breast lesions were included in the non-scanned missed tissue neither from large resection sections nor core biopsies. That is because WSI captures pre-scans of the whole slide at low resolution, and only tissue detected on the pre-scan is then scanned at high magnification. All previously mentioned breast lesions are easily detected in the low-resolution image and completely scanned on the digital image. None of the studied core biopsies revealed missing tissue which is quite reassuring for the pathologist in reporting during routine clinical practice. This may be due to the nature of core biopsies as it is basically punched from mass forming lesions with less fat content. Missing tissue did not affect the final diagnosis of the cases nor the tumor grade or stage. Although, 12% of cases revealed a reduction of distance to actual margin, none of the cases was misidentified as having a positive margin (tumor on ink)^11^, and no subsequent re-excision was carried out.

Although these findings could reassure the majority of pathologists that using WSIs in histopathology reporting is optimal, the risk of missing specific lesions that arise in adipose soft tissue like liposarcoma needs further investigation. This support the hypothesis that the guidelines of using WSIs in routine pathology reporting should be individualized based on several factors as mentioned above in addition to tissue type. Considerations of such specific limitations without generalization will help in faster implementation of the technology.

The frequency of missing tissue in WSI of IHC stained sections was higher than the H&E WSIs, which is expected as IHC sections usually thinner and using hematoxylin only as a counterstain reduce the tissue contrast thus fatty tissue in IHC sections tends to be paler and fainter. Nevertheless, the main missed tissue was fat which did not affect the assessment of the IHC biomarkers. IHC sections are more liable to tissue folding/loosing during staining especially from antigen retrieval method. This uneven tissue surface usually affects the quality of the acquired images with areas out of focus. This could affect the assessment of IHC biomarkers, not due to missed tissue but due to difficulty in evaluating such hazy areas. Therefore, standard QC measures focusing on the overall quality of the slides including tissue processing, sectioning quality, and staining quality should be followed in addition to QC of the scanning process to ensure the highest quality WSI of the IHC stained slides.

Using an overview image (macro-image) could help pathologists detect missing tissue to determine if the WSI would be enough for diagnosis, especially if the missing tissue proportion is low or they need to return to glass slides if the missed tissue is peripherally located and would affect surgical margin measurement.

In this study, we compared between the frequency of missing tissue and the image quality within the first 3 months of digitalization of the department and the following 3 months to assess the impacts of early implementation of the technology and full application of the QC measures in routine practice. There was an improvement in the image quality and reduction of WSI failure rates following the initial implementation period. The image failure was categorized into tissue related such as folding and scores, faint and blurred WSI, scanning errors i.e., longitudinal scores, incomplete scanning or out of focus areas. Issues related to slide preparation and microtomy was solved by proper calibration of microtomes and changing tissue thickness from 2.5 to 4 μm and re-staining of faint slides^14^. Proper cleaning of slides as well as avoiding extensions of tissue fragments beyond the coverslips minimizqes missing parts of the tissue near the edges of the slide^7^. Other image failure causes were poor quality scan, and this was solved by rescanning the slides. Our findings indicated that QC measures were successful to reduce the image failure rates. However, the missing tissue frequency was not significantly improved over time even with the adoption of these strict QC measures. This could be explained by the nature of the tissue and the scanner performance and its ability to detect low density and faint tissue rather the quality of the slides’ preparation.

Although strict QC measures can reduce scanned image failure rates, they may add costs to the digitizing process in terms of staff, time, and that the WSI can be considered as an additional financial burden on pathology laboratories. Unlike digitization of radiology, WSI technology in pathology is considered as an additional rather than a replacement technology. WSI will not be able to create digitalized WSI without the creation of the glass slides. However, we need to highlight that the return on investment measures in WSI in pathology are mainly related to changing the geographically fixed workload of glass slide reporting to a fully flexible workflow which can be delivered electronically. Just as with mail communications, this change has multiple benefits and efficiencies to be exploited, including sharing workload between multiple pathologists, centers and regions, rapid and convenient peer review of cases, reporting remotely from the host laboratory, 24-h working and, easy access to archived material. More importantly the promise of AI algorithms that can result in a paradigm shift in the field of pathology^15,16^ can only be delivered to a digitized workflow.

In our study, a negative correlation between the frequency of missing tissue and both the scanning time and the image file size was observed. Also, scanners without human input during scanning had a shorter scanning time, but the highest frequency of missing tissue as it tends to ignore faint tissue during scanning to speed up the process.

In conclusion, missing tissue on breast WSIs is observed with variable frequency. Fatty tissue is frequently missed but is of little diagnostic consequence. Improving scanning algorithms and increasing the tolerance towards slide margins can potentially improve the performance and user confidence in the technology. Balancing between WSIs quality and scanning time/image file size should be considered. Similar to glass slide preparation, adequate QC measures can reduce WSI failure rates and diminish the potential clinical implications of missing tissue on WSIs.

## Supplementary information

Supplementary table

## Data Availability

All data used in this study are available and can be accessed upon reasonable request.
