# Supplementary material for: Potential quality pitfalls of digitalized whole slide image of breast pathology in routine practice
Source: Mod Pathol. 2021 Dec 27;35(7):903–10. doi: 10.1038/s41379-021-01000-8 (PMC8711290; doi:10.1038/s41379-021-01000-8)
Supplement: Supplementary file 1 — Supplementary table [file 41379_2021_1000_MOESM1_ESM.docx]

**Supplementary Table**: Detailed proportion of missing tissue among all studied cases in cohort 1 (scanned in the routine diagnostic practice)

| Proportion of missing tissue | Number of cases | Percent out of total studied cases | Percent out of missing tissue cases | Cumulative Percent |  |
| --- | --- | --- | --- | --- | --- |
| 1% | 760 | 3.5 | 17.8 | 17.8 | |
| 2% | 401 | 1.8 | 9.4 | 27.2 | |
| 3% | 191 | 0.9 | 4.5 | 31.7 | |
| 4% | 106 | 0.5 | 2.5 | 34.2 | |
| 5% | 615 | 2.8 | 14.4 | 48.5 | |
| 6% | 21 | 0.1 | .5 | 49.0 | |
| 7% | 85 | 0.4 | 2.0 | 51.0 | |
| 8% | 64 | 0.3 | 1.5 | 52.5 | |
| 10% | 572 | 2.6 | 13.4 | 65.8 | |
| 12% | 43 | 0.2 | 1.0 | 66.8 | |
| 15% | 250 | 1.2 | 5.9 | 72.8 | |
| 17% | 21 | 0.1 | .5 | 73.3 | |
| 20% | 358 | 1.6 | 8.4 | 81.7 | |
| 30% | 250 | 1.2 | 5.9 | 87.6 | |
| 35% | 64 | 0.3 | 1.5 | 89.1 | |
| 40% | 127 | 0.6 | 3.0 | 92.1 | |
| 50% | 212 | 1.0 | 5.0 | 97.0 | |
| 60% | 85 | 0.4 | 2.0 | 99.0 | |
| 70% | 43 | 0.2 | 1.0 | 100.0 | |
| Total cases with missing tissue | **4268** | **19.4** | **100.0** |  | |
| Total studied cases | **22000** | **80.6** |  |  | |
